# Asymptomatic Cardiotoxicity After High‐Dose Anthracycline Treatment in Sarcoma Patients Assessed by Biomarkers and Echocardiography

**DOI:** 10.1002/ijc.70620

**Published:** 2026-06-30

**Authors:** Elissa A. S. Polomski, Frank M. Speetjens, J. Wouter Jukema, Julius C. Heemelaar, Michiel A. J. van de Sande, Hans Gelderblom, M. Louisa Antoni

**Affiliations:** ^1^ Department of Cardiology, Heart Lung Center Leiden University Medical Center Leiden the Netherlands; ^2^ Department of Medical Oncology Leiden University Medical Center Leiden the Netherlands; ^3^ Netherlands Heart Institute Utrecht the Netherlands; ^4^ Department of Orthopedic Surgery Leiden University Medical Center Leiden the Netherlands

**Keywords:** biomarkers, cardio‐oncology, cardiotoxicity‐related cardiac dysfunction, echocardiography, sarcoma

## Abstract

Treatment of sarcoma includes high‐dose anthracyclines, which can cause cardiotoxicity. This study assesses the correlation of cardiac biomarkers and echocardiographic parameters on asymptomatic cardiotoxicity in sarcoma patients treated with anthracyclines. Consecutive patients diagnosed with sarcoma and treated with anthracyclines were included in this retrospective cohort study using registry data. Echocardiography was performed before start of anthracycline chemotherapy (baseline), after completing treatment (short‐term follow‐up), and 6–12 months after termination of treatment (long‐term follow‐up). The primary endpoint was the occurrence of asymptomatic cardiotoxicity at short‐term follow‐up. We included 44 patients (29.6% female) with a median age of 28.9 [22.2–38.7] years. Baseline left ventricular ejection fraction (LVEF) and global longitudinal strain (GLS) decreased significantly from 58.4% ± 5.0% to 54.2% ± 9.3% (*p* = 0.013) and −19.1% ± 2.2% to −17.2% ± 3.5% at short‐term follow‐up (*p* = 0.002), respectively. After Cycle 1, 86.5% of the patients showed NT‐proBNP ≥ 125 ng/L compared to 3.9% at baseline (*p* < 0.001), and after completion of therapy, 65.5% had elevated cardiac Troponin T (cTnT) ≥ 14 ng/L compared to 11.5% at baseline (*p* = 0.003). Asymptomatic cardiotoxicity was observed in 30 patients (68.2%). Mean follow‐up was 2.4 [1.5–2.9] years. cTnT ≥ 14 ng/L after Cycle 4 was associated with cardiotoxicity (HR: 3.75 [1.28–11.0], *p* = 0.016), and NT‐proBNP per 100 units increase after Cycle 1 was associated with an increased risk of mortality (HR: 1.36 [1.004–1.85], *p* = 0.047). Sarcoma patients treated with high‐dose anthracyclines are at increased risk of developing cardiac dysfunction as assessed by LVEF and GLS. cTnT and NT‐proBNP levels increase significantly during treatment and can be valuable markers to improve the risk stratification of patients treated with high‐dose anthracyclines.

## Abstract (highlights)

What's new?

Current guidelines recommend measuring cardiac biomarkers before and during anthracycline chemotherapy for early detection of myocardial injury and subsequent cardiotoxicity. In this retrospective single‐center study, patients with sarcoma treated with high‐dose anthracyclines showed an increased risk of left ventricular dysfunction, as assessed by left ventricular ejection fraction and global longitudinal strain. Cardiac biomarkers rose significantly after the first cycle of anthracyclines and remained elevated during treatment. Elevated cardiac troponin T was associated with increased risk of asymptomatic cardiotoxicity and NT‐proBNP with increased risk of mortality. The findings underscore the need for prompt monitoring of cardiac biomarkers in this high‐risk population.

## Graphical abstract

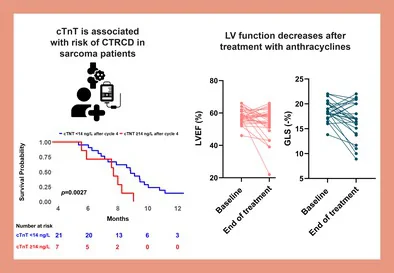

**Abbreviations**
cTnT: cardiac troponin T
CTRCD: cardiotoxicity‐related cardiac dysfunction
GLS: global longitudinal strain
HF: heart failure
IVADo/IVA: ifosfamide/vincristine/d‐actinomycin/doxorubicin and ifosfamide/vincristine/d‐actinomycin
LAVi: left atrial volume index
LVEDV: left ventricular end‐diastolic volume
LVEF: left ventricular ejection fraction
LVESV: left ventricular end‐systolic volume
MAP/AP: methotrexate/doxorubicin/cisplatin and doxorubicin/cisplatin
NP: natriuretic peptides
NT‐proBNP: N‐terminal prohormone of brain natriuretic peptide
ROS: reactive oxygen species
RV: right ventricular
TAPSE: tricuspid annular plane systolic excursion
VDC‐IE: vincristine/doxorubicin/cyclophosphamide and ifosfamide/etoposide

## 1 Introduction

Primary bone tumors account for less than 1% of the yearly cancer diagnoses [1]. The most common primary malignant bone tumor is osteosarcoma, followed by Ewing sarcoma [2]. Treatment usually consists of surgical excision of the tumor in combination with (neo)adjuvant chemotherapy, including high‐dose anthracyclines [3]. Anthracyclines are well‐known for their dose‐dependent risk of cardiotoxic side effects and increase the risk of cardiac dysfunction and mortality [4, 5]. The risk of congestive heart failure is already present during treatment, but increases over time and still is observed more than 15 years after anthracycline therapy, probably due to irreversible damage through cumulative DNA damage by reactive oxygen species (ROS) [6, 7]. Therefore, monitoring cardiac function using echocardiography is recommended before, during and after anthracycline chemotherapy [8]. Research has shown that global longitudinal strain (GLS) and left ventricular ejection fraction (LVEF) can identify cancer survivors at high risk of developing cardiotoxicity and cardiac events by early subclinical cardiac dysfunction [9, 10]. In prior guidelines, cardiotoxicity was primarily defined by echocardiography, without incorporating cardiac biomarkers and no explicit distinction was made between the severity levels. GLS was not included in all definitions and the threshold LVEF was less strict, ranging between 50% and 55% [11].

The more recent ESC guidelines on cardio‐oncology have expanded this definition, incorporating a rise in cardiac biomarkers and defining three categories of asymptomatic cardiotoxicity based on LVEF, GLS, and cardiac biomarkers. These guidelines state that it should be considered to measure natriuretic peptides (NP) and cardiac troponin (cTn) before and during anthracycline chemotherapy for early detection of myocardial injury and subsequent cardiotoxicity [8]. Studies observed significantly increased cTnT levels posttreatment compared to pretreatment and an association between increased plasma levels and the risk of cardiotoxicity [12, 13]. Results on the association between NP and anthracycline therapy are scarce, but small studies have shown that levels exceeding a predefined cut‐off value were more common posttreatment [14, 15].

However, in the high‐risk population of sarcoma patients, there is a gap in evidence considering routinely measurement of serum cTnT and NP and their relation to changes in echocardiographic parameters and (asymptomatic) cardiac dysfunction. Therefore, this study aims to apply the contemporary ESC criteria in an adult sarcoma population and assess the correlation of cTnT and N‐terminal prohormone of brain natriuretic peptide (NT‐proBNP) before and during anthracycline therapy and echocardiographic parameters on asymptomatic cardiotoxicity‐related cardiac dysfunction (CTRCD).

## 2 Methods

### 2.1 Study Design and Population

Consecutive patients who were diagnosed with osteosarcoma, Ewing sarcoma, rhabdomyosarcoma, leiomyosarcoma of bone or undifferentiated pleomorphic sarcoma of bone treated at the Leiden University Medical Center, a tertiary referral center for sarcoma, since 2019 were enrolled in an ongoing registry. This is a single‐center retrospective cohort study based on a prospective sarcoma registry. Our current study retrospectively examines the available data up to 12 months of follow‐up. Patients who presented with sarcoma between 2019 and 2023 were included if they were treated with anthracyclines and if an echocardiogram was available before start of anthracycline chemotherapy. Patients were excluded if they had a prior cancer diagnosis treated with chemotherapy, if chemotherapy was not completed or if they had a history of ischemic heart disease. The process of patient inclusion is shown in Figure 1. All baseline echocardiograms were performed within 1 month before start of anthracycline therapy. Hypertension, hypercholesterolemia, and diabetes were defined based on a documented medical history of these conditions. Smoking was defined as current or past smoking. All patients with osteosarcoma, leiomyosarcoma or undifferentiated pleomorphic sarcoma were treated according to the EURAMOS [16] protocol with Methotrexate/Doxorubicin/Cisplatin and Doxorubicin/Cisplatin (MAP/AP), patients with Ewing sarcoma according to the EURO EWING 2012 protocol [17] with Vincristine/Doxorubicin/Cyclophosphamide and Ifosfamide/Etoposide (VDC‐IE) and patients with rhabdomyosarcoma were treated according to the IVADo/IVA scheme with ifosfamide/vincristine/d‐actinomycin/doxorubicin and ifosfamide/vincristine/d‐actinomycin (IVADo/IVA) according to the RMS 2005 protocol [18].

**FIGURE 1:**
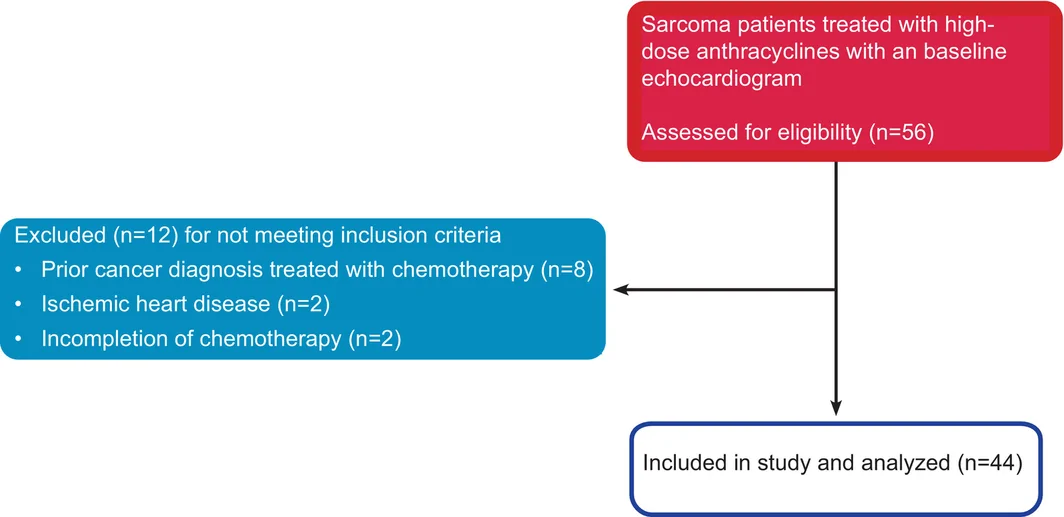
STROBE diagram. This figure shows the process of patient inclusion.

### 2.2 Data Collection

Demographic patient data, medication, medical history, laboratory values, oncological characteristics, and cardiovascular history and risk factors were collected through the electronic pharmacy records (HiX Version 6.3 Chipsoft and EPD‐Vision, the Netherlands). Laboratory values were collected at baseline, before the first administration of anthracyclines. After each anthracycline cycle, cTnT and NT‐proBNP serum plasma levels were collected if available.

### 2.3 Echocardiography

Patients underwent echocardiographic examination at different timepoints: before start of anthracycline chemotherapy (baseline), after completing treatment (short‐term follow‐up), and 6 months–1 year after termination of treatment (long‐term follow‐up).

Data acquisition of echocardiographic images was carried out using 3.5 MHz or M5S transducers and images were obtained in the left lateral decubitus position using the Vivid 7, E9, and E95 systems (GE Vingmed, Horten, Norway). In addition to the standard color and 2D images, pulsed wave and continuous wave Doppler images were acquired according to the guidelines [19] with dedicated software (Echopac V.240; GE Vingmed, Horten, Norway). All reported echocardiographic parameters are conventional measurements that are frequently used in clinical practice. All images were stored offline for analysis. Previous studies from our center have reported good intra‐ and interobserver variability [20].

The left ventricular (LV) end‐diastolic volume (LVEDV) and the LV end‐systolic volume (LVESV) were measured by using the apical two‐ and four chamber view. The LVEF was calculated by using the Simpon's biplane method and the modified Simpson method was used to calculate the left atrial volume index (LAVi). Two‐dimensional speckle‐tracking strain analysis (EchoPAC V.240) was performed to assess LV GLS as a measure of the LV systolic function. Deformation of the myocardium was analyzed by frame‐to‐frame tracking of the movement of acoustic markers. The endocardial border of the left ventricle was traced in apical two‐, three‐ and four‐chamber views and regions of interest were adjusted manually. By taking the average of the different longitudinal strain values of the different apical views, the LV GLS was calculated automatically. A more negative GLS value defines better deformation of the LV myocardium as shortening of the LV myocardium is reflected by a negative GLS value. LV systolic dysfunction was defined as an LVEF < 50% or an LV GLS > −16% [21]. To assess LV diastolic function, peak early (E) and late (A) diastolic velocities were acquired by pulsed‐wave Doppler recordings of the transmitral flow. The lateral and septal e′ were obtained by mitral annulus Doppler imaging and averaged for the calculation of E/e′ as the reflection of filling pressures of the left ventricle [22]. Diastolic dysfunction was graded according to the following parameters: LAVi ≥ 34 mL/m^2^, lateral e′ < 10 cm/s, and septal e′ < 8 cm/s [22], see Figure S1. Tricuspid annular plane systolic excursion (TAPSE) and S′ were measured to assess right ventricular (RV) systolic function. The cut‐off values for TAPSE and RV S′ were < 17 mm and < 9.5 cm/s, respectively [19].

### 2.4 Asymptomatic Cardiotoxicity‐Related Cardiac Dysfunction and Cardiac Biomarkers

In consistence with the guidelines [23], asymptomatic CTRCD was divided into three categories: severe, moderate, and mild asymptomatic CTRCD. In short, severe CTRCD is defined as a drop in LVEF to < 40%, moderate CTRCD as a drop in LVEF to 40%–49% with or without a drop in GLS and/or a rise in cardiac biomarkers, and mild CTRCD as an LVEF ≥ 50% with either a drop in GLS or rise in cardiac biomarkers. The full definitions of these categories are presented in Table S1. In addition, an imaging‐defined CTRCD endpoint was evaluated, based exclusively on changes in LVEF and/or GLS, without inclusion of biomarker criteria. The imaging‐defined CTRCD endpoint was included to assess the association between early biomarker changes and subsequent structural cardiac dysfunction while avoiding potential incorporation bias due to overlap between biomarker‐based predictors and outcome definition. For clarity, we will refer to CTRCD with the term cardiotoxicity throughout the manuscript. Cardiac biomarkers were collected at baseline and after each cycle of anthracyclines. The cut‐off value for NT‐proBNP was defined as ≥ 125 ng/L. For cTnT, gender specific cut‐off values of ≥ 9 and ≥ 14 ng/L were used for female and male patients, respectively. In addition, a gender neutral cTnT cut‐off value of ≥ 14 ng/L was applied [23, 24]. A rise in biomarkers was defined as a rise between baseline and any timepoint to a value above the predefined cut‐off value in the presence of a normal range at baseline or a rise beyond the biological and analytical variation of the assay used, according to the guidelines [23].

### 2.5 Endpoint

The primary endpoint was the occurrence of any grade of asymptomatic cardiotoxicity at short‐term follow‐up. The secondary study outcome was all‐cause mortality.

### 2.6 Statistical Analysis

Depending on the normality of distribution, continuous variables are presented as mean ± standard deviation or median with interquartile range (IQR). Categorical variables are expressed as counts with percentages. The Chi‐square test or Fisher's exact test was used to compare binary and categorical variables, as appropriate. Echocardiographic parameters and cardiac biomarkers were analyzed using linear or generalized linear mixed‐effects models depending on the outcome type. Continuous outcomes were analyzed with linear mixed‐effects models and binary outcomes with binomial GLMMs using a logit link function. Time was treated as a categorical variable to compare all available timepoints and a random intercept for each patient was included to account for repeated measurements within individuals. A compound symmetry covariance structure was used. No imputation was performed. Post hoc pairwise comparisons between timepoints were performed using estimated marginal means. Bonferroni adjustment was applied to correct for multiple testing. Sensitivity analyses were conducted using complete‐case data and are reported only for variables where the results differed from the mixed‐effects model. For each pairwise comparison between timepoints, only patients with measurements available at the relevant timepoints were included. Cox proportional hazard regression analysis was performed to assess the association between cardiac biomarkers and echocardiographic parameters on the primary and secondary study outcomes. A two‐sided *p*‐value of < 0.05 was considered as statistically significant. All analyses were performed in STATA version 17.0 (StataCorp 2021, College Station, Texas, USA) and in R Version 4.3.1 (R Core Team 2023, R Foundation for Statistical Computing, Vienna, Austria).

## 3 Results

### 3.1 Baseline Characteristics

We included 44 patients with a median age of 28.9 [22.2–38.7] years at diagnosis of sarcoma of whom 13 (29.6%) were female. Cardiovascular risk factors were present as follows: one patient (2.3%) had hypertension, one patient (2.3%) diabetes mellitus type II, 11 patients (25.0%) were (ex‐)smokers and one patient (2.3%) had a history of congestive heart disease. The majority of the patients were diagnosed with Ewing sarcoma (*n* = 22, 50.0%), 17 patients with osteosarcoma (38.6%), 2 patients with rhabdomyosarcoma (4.5%), 1 patient with leiomyosarcoma (2.3%) and 2 patients with undifferentiated pleomorphic sarcoma (4.5%). Distant metastases at baseline were present in 7 patients (15.9%). Patients were treated with different schemes, according to their type of sarcoma. Each of the 44 patients received anthracyclines, with a median dose of 375 [375–450] mg/m^2^. Baseline characteristics of our study population are presented in Table 1.

**TABLE 1:** Baseline characteristics.

	Total (*n* = 44)	No CTRCD (*n* = 14)	CTRCD (*n* = 30)	*p*
Female, *n* (%)	13 (29.5%)	4 (28.6%)	9 (30.0%)	1.000
BMI, mg/m^2^	24.3 [20.9–29.5]	27.2 [20.8–29.6]	23.4 [21.1–29.3]	0.392
Age at diagnosis, yrs	28.9 [22.2–38.7]	28.8 [22.3–45.5]	29.3 [22.0–37.4]	0.614
Medical history				
Hypertension	1 (2.3%)	1 (7.1%)	0 (0.0%)	0.318
Hypercholesterolemia	0 (0.0%)	0 (0.0%)	0 (0.0%)	
DM II	1 (2.3%)	1 (7.1%)	0 (0.0%)	0.318
Smoking	11 (25.0%)	3 (21.4%)	8 (26.7%)	1.000
IHD	0 (0.0%)	0 (0.0%)	0 (0.0%)	
CHD	1 (2.3%)	1 (7.1%)	0 (0.0%)	0.318
Cardiac medication	2 (4.5%)	2 (14.3%)	0 (0.0%)	0.096
ACEi	1 (50.0%)	1 (50.0%)	0 (0.0%)	0.318
Statin	1 (50.0%)	1 (50.0%)	0 (0.0%)	0.318
CCB	1 (50.0%)	1 (50.0%)	0 (0.0%)	0.318
Type of sarcoma				0.052
Osteosarcoma	17 (38.6%)	8 (57.1%)	9 (30.0%)	0.085
Ewing Sarcoma	22 (50.0%)	4 (28.6%)	18 (60.0%)	0.104
Rhabdomyosarcoma	2 (4.5%)	2 (14.3%)	0 (0.0%)	0.096
Leiomyosarcoma	1 (2.3%)	0 (0.0%)	1 (3.3%)	1.000
Undifferentiated pleiomorphic sarcoma	2 (4.5%)	0 (0.0%)	2 (6.7%)	0.1.000
Metastasis	7 (15.9%)	2 (14.3%)	5 (16.7%)	1.000
Cancer treatment				
Surgery	39 (88.6%)	11 (78.6%)	28 (93.3%)	0.151
Anthracyclines	44 (100.0%)	14 (100.0%)	30 (100.0%)	
Anthracycline dose, mg/m^2^	375.0 [375.0–450.0]	337.5 [150.0–375.0]	375.0 [375.0–450.0]	0.017
Chemotherapy				0.034
MAP/AP	20 (45.5%)	8 (57.1%)	12 (40.0%)	0.287
VDC‐IE	22 (50.0%)	4 (28.6%)	18 (60.0%)	0.104
LVADo/IVA	2 (4.5%)	2 (14.3%)	0 (0.0%)	0.096
Radiotherapy	16 (36.4%)	4 (28.6%)	12 (40.0%)	0.521
Involved node	15 (34.1%)	3 (21.4%)	12 (40.0%)	0.314
Thoracic	1 (2.3%)	1 (7.1%)	0 (0.0%)	0.318
Radiotherapeutic dose (Gy)	54.0 [50.4–95.8]	77.0 [44.0–88.3]	54.0 [50.4–99.0]	1.000
Laboratory				
Hb, mmol/L	8.2 [7.6–8.9]	8.0 [7.4–9.2]	8.3 [7.6–8.9]	0.830
Leukocytes, ×10^9^/L	7.4 [6.0–9.2]	7.1 [6.1–9.2]	7.5 [6.0–9.1]	0.960
Trombocytes, ×10^9^/L	257.5 [225.5–324.5]	259.5 [237.0–345.0]	254.5 [222.0–290.0]	0.457
Creatinine, umol/L	65.0 [56.0–73.0]	64.5 [58.0–73.0]	65.0 [56.0–73.0]	0.980
CRP, mg/L	1.8 [1.8–30.4]	1.8 [1.8–1.8]	16.1 [1.8–30.4]	0.480
Glucose, mmol/L	5.3 [5.0–5.8]	5.3 [5.0–5.7]	5.2 [5.0–5.9]	0.898
LDH, U/L	188.0 [161.0–235.0]	205.5 [180.0–233.0]	180.0 [160.0–235.0]	0.378
Troponine T, ng/L	6.0 [1.0–8.0]	1.0 [1.0–6.0]	6.0 [1.0–9.0]	0.122
NT‐proBNP, ng/L	32.4 [17.1–66.1]	69.0 [17.1–85.6]	27.1 [16.7–48.6]	0.112

Abbreviations: ACEi, Angiotensin‐converting enzyme inhibitor; BMI, body mass index; CCB, calcium channel blocker; CHD, congestive heart disease; DM II, Diabetes Mellitus Type II; Gy, Gray; IHD, ischemic heart disease; LVADo/Iva, ifosfamid/vincristine/d‐actinomycine/doxorubicine and ifosfamide/vincristine/d‐actinomycine; MAP/AP, Mehotrexate/Doxorubicin/Cisplatin and Doxorubicin/Cisplatin; NT‐proBNP, N‐terminal prohormone of brain natriuretic peptide; VDC‐IE, Vincristin/Doxorubicin/Cyclophosfamide and Ifosfamid/Etoposid.

### 3.2 Echocardiographic Parameters

Time between baseline echocardiogram and short‐term follow‐up was 7.3 ± 2.1 months and between baseline and long‐term follow‐up, this was 1.34 ± 0.41 years. Echocardiography at baseline was performed in all (*n* = 44) patients, at short‐term follow‐up in 32 patients and at long‐term follow‐up in 23 patients. Differences in echocardiographic parameters between the timepoints are shown in Table 2.

**TABLE 2:** Echocardiographic parameters.

	Baseline	Short‐term follow‐up	*p*	Long‐term follow‐up	*p* *
LVEF (%)	58.4 ± 5.0	54.2 ± 9.3	0.013	52.2 ± 6.0	< 0.001
GLS (%)	−19.1 ± 2.2	−17.2 ± 3.5	0.002	−17.1 ± 3.0	0.006
LVESV (mL)	46.9 ± 13.2	54.0 ± 18.4	0.078	51.9 ± 10.0	1.000
LVEDV (mL)	112.2 ± 30.1	117.9 ± 30.8	01.000	109.9 ± 26.3	0.788
Diastolic dysfunction	0 (0%)	2 (8.3%)	1.000	1 (4.8%)	1.000
E/A	1.44 ± 0.47	1.65 ± 1.10	0.551	1.49 ± 0.50	1.000
E/e′	5.15 ± 1.10	5.43 ± 1.63	1.000	4.58 ± 0.84	1.000
Deceleration time (ms)	211.2 ± 45.3	185.4 ± 63.2	0.341	157.9 ± 37.5	0.039
LAVI (mL/m^2^)	25.2 ± 7.0	25.0 ± 7.9	1.000	23.5 ± 5.7	0.109
Peak E wave velocity (m/s)	77.8 ± 11.4	72.1 ± 18.2	0.148	68.3 ± 18.5	< 0.001
Peak A wave velocity (m/s)	57 ± 12.5	52.0 ± 16.2	0.215	50.3 ± 14.0	0.261
Septal e′ velocity (cm/s)	13.9 ± 2.4	11.8 ± 3.1	0.010	11.9 ± 2.7	0.083
Lateral e′ velocity (cm/s)	17.2 ± 3.8	15.4 ± 4.0	0.0497	16.0 ± 3.4	0.134
TAPSE (mm)	23.0 ± 3.1	21.7 ± 3.3	0.339	21.6 ± 3.2	0.191
RV S′ (cm/s)	14.4 ± 2.5	13.0 ± 3.0	0.304	13.0 ± 2.2	0.295
RV strain (%)	−25.8 ± 4.5	−21.6 ± 4.5	0.438	−21.1 ± 4.4	1.000

Abbreviations: GLS, global longitudinal strain; LAVI, left atrial volume index; LVEDV, left ventricular end‐diastolic volume; LVEF, left ventricular ejection fraction; LVESV, left ventricular end‐systolic volume; RV, right ventricle; TAPSE, tricuspid annular plane systolic excursion.

* This *p*‐value represents the difference between T1 and T2.

#### 3.2.1 Left Ventricular Function

The LVEF of the whole cohort was 58.4% ± 5.0% at baseline and decreased significantly over time to 54.2% ± 9.3% (*p* = 0.013) at short‐term follow‐up and 52.2% ± 6.0% at long‐term follow‐up (*p* < 0.001). The change in mean LVEF from short‐term follow‐up to long‐term follow‐up was not statistically significant (*p* = 0.662). A reduced LVEF < 50% was observed in 2 patients (4.6%) at baseline, 5 patients (16.7%) at short‐term follow‐up (*p* = 0.046), and 6 patients (30.0%) at long‐term follow‐up (*p* = 0.004). In six patients (19.4%) the LVEF dropped by ≥ 10% between baseline and short‐term follow‐up. Baseline GLS was −19.1% ± 2.2% and decreased significantly to −17.2% ± 3.5% at short‐term follow‐up (*p* = 0.002) and −17.1% ± 3.0% (*p* = 0.006) at long‐term follow‐up. Between short‐term follow‐up and long‐term follow‐up, GLS stayed constant (*p* = 1.000). A declined GLS of > −16% was observed in 2 patients (4.6%) at baseline, 7 patients (23.3%) at short‐term follow‐up (*p* = 0.402), and 6 patients (30.0%) at long‐term follow‐up T2 (*p* = 1.000). None of the patients showed a drop in GLS of > 15% during follow‐up. Septal velocity also declined significantly between baseline and short‐term follow‐up from 13.9 ± 2.4 cm/s to 11.9 ± 3.1 cm/s (*p* = 0.010). None of the patients had any grade of diastolic dysfunction at baseline, but two patients developed grade 1 diastolic dysfunction after chemotherapy (*p* = 1.000).

#### 3.2.2 Right Ventricular Function

RV strain remained constant between baseline short‐term follow‐up (*p* = 0.438) as well as long‐term follow‐up (*p* = 1.000). Two patients showed a RV S′ lower than the threshold value of < 9.5 cm/s at baseline and two patients developed this after treatment with anthracyclines. TAPSE showed no significant change from baseline to short‐term follow‐up (23.0 ± 3.1 mm to 21.7 ± 3.3 mm, *p* = 0.339) and long‐term follow‐up (*p* = 0.191). At baseline, one patient had a TAPSE below reference level (< 17.5 mm) and in another patient TAPSE decreased from 21 to 16 mm after treatment.

### 3.3 Cardiac Biomarkers

#### 3.3.1 NT‐proBNP

Baseline NT‐proBNP was available for 26 patients with a median value of 32.4 [17.1–66.1] ng/L. The median highest measured NT‐proBNP level at any timepoint was 547.5 [366.6–862.3] ng/L. An overview of available and median NT‐proBNP values at the different timepoints is shown in Figure 2. In all patients, NT‐proBNP rose after one cycle of anthracyclines compared to baseline as can be observed in Figure 2. NT‐proBNP increased significantly between baseline and Cycle 1 from 27.8 [16.7–49.6] ng/L to 323.0 [180.5–686.2] ng/L (*p* < 0.001) and continued to be significantly elevated compared to baseline during the other cycles (*p* < 0.001) (Figure 2). However, after Cycle 1, NT‐proBNP levels did not show any further significant rise. According to prior literature, a cut‐off value of ≥ 125 ng/L was employed to define elevated NT‐proBNP. At baseline, only one patient (3.9%) showed NT‐proBNP ≥ 125 ng/L, whereas after Cycle 1, this significantly increased to 32 patients (86.5%, *p* = 0.029). The difference in number of patients with NT‐proBNP ≥ 125 ng/L after Cycle 1 remained significantly higher compared to baseline during all subsequent cycles of chemotherapy (Figure 3).

**FIGURE 2:**
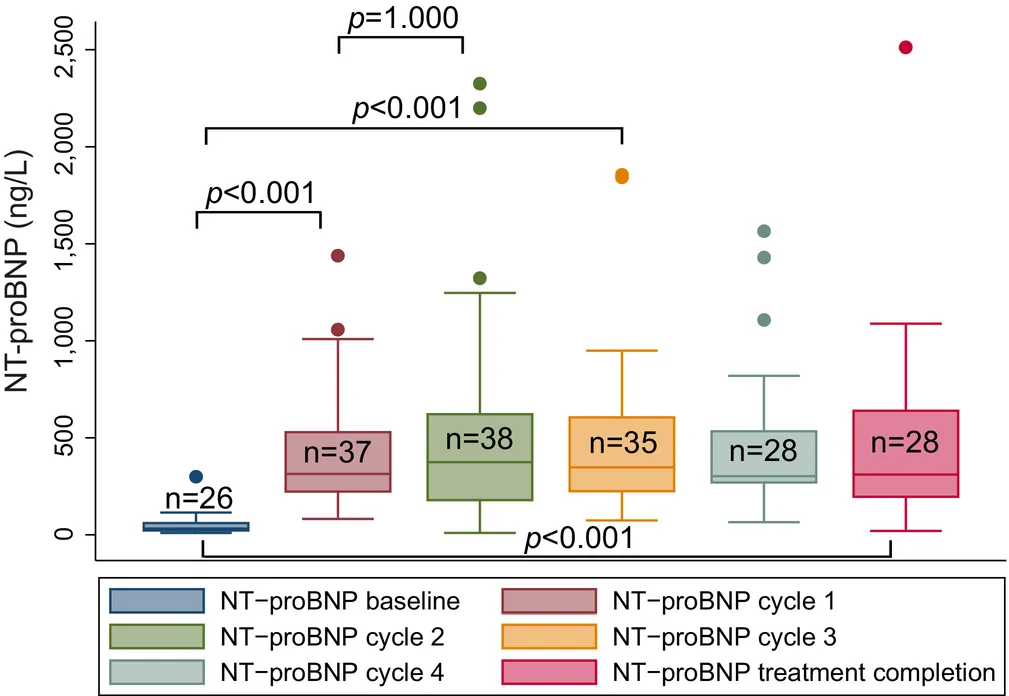
Increase in NT‐proBNP between all timepoints. This figure shows the differences in NT‐proBNP between the timepoints. NT‐proBNP increases significantly between baseline and Cycle 1 (*p* < 0.001) and remains significantly elevated during the other cycles compared to baseline. No differences between the other timepoints were observed in the increase of NT‐proBNP.

**FIGURE 3:**
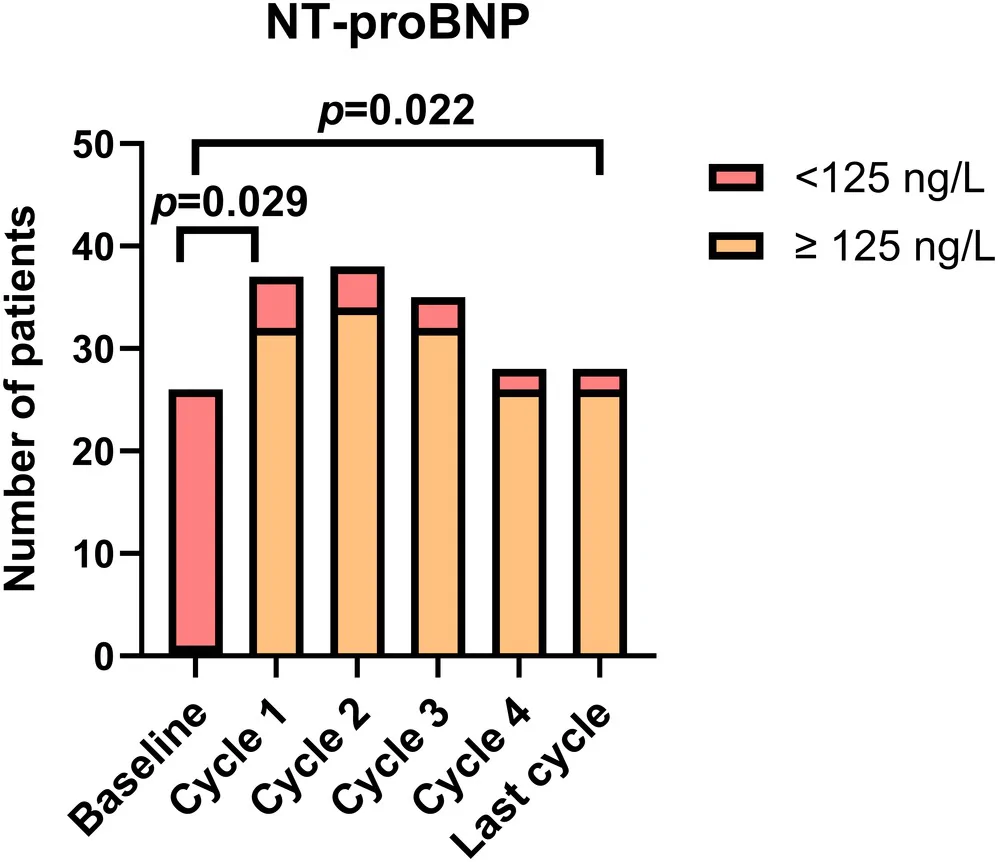
Elevated NT‐proBNP. This figure presents the number of patients with elevated NT‐proBNP (≥ 125 ng/L) at all timepoints. The number of patients increases significantly between baseline and Cycle 1 and stays significantly elevated during treatment. Last cycle refers to treatment completion.

#### 3.3.2 Cardiac Troponin T (cTnT)

cTnT values were available for 26 patients at baseline with a median of 6 [1–8] ng/L. Median cTnT values at the different timepoints are presented in Figure 4. Maximum median cTnT was 16 [11–24.5] ng/L. cTnT increased significantly to 14 [10–20] ng/L (*p* = 0.006) between baseline and completion of therapy as well as between completion of therapy and various other cycles as shown in Figure 4. A gender neutral cut‐off value of ≥ 14 ng/L was used and at baseline, three patients (11.5%) had elevated cTnT levels. Although no significant rise was observed in elevated cTnT after the first 4 cycles, 19 patients (65.5%) showed an elevated cTnT after completion of therapy compared to baseline (*p* = 0.004) (Figure 5A). Gender neutral cTnT was also significantly elevated after completion of therapy compared to Cycle 1 (*p* < 0.001), Cycle 2 (*p* < 0.001), Cycle 3 (*p* = 0.002), and Cycle 4 (*p* = 0.044), see Figure 5A. As the gender distribution is skewed in our population (29.6% female), we also assessed the association between the cycles for the combined gender specific cut‐off values of ≥ 9 ng/L (f) and ≥ 14 ng/L (m) (Figure 5B). Three patients (11.5%) had gender specific elevated cTnT at baseline, which significantly increased to 20 patients (69.0%) after completion of therapy (*p* = 0.002) (Figure 5B). In line with gender neutral cTnT, gender specific cTnT was also significantly elevated after completion of therapy compared to Cycle 1 (*p* < 0.001), Cycle 2 (*p* < 0.001), Cycle 3 (*p* = 0.005), and Cycle 4 (*p* = 0.047).

**FIGURE 4:**
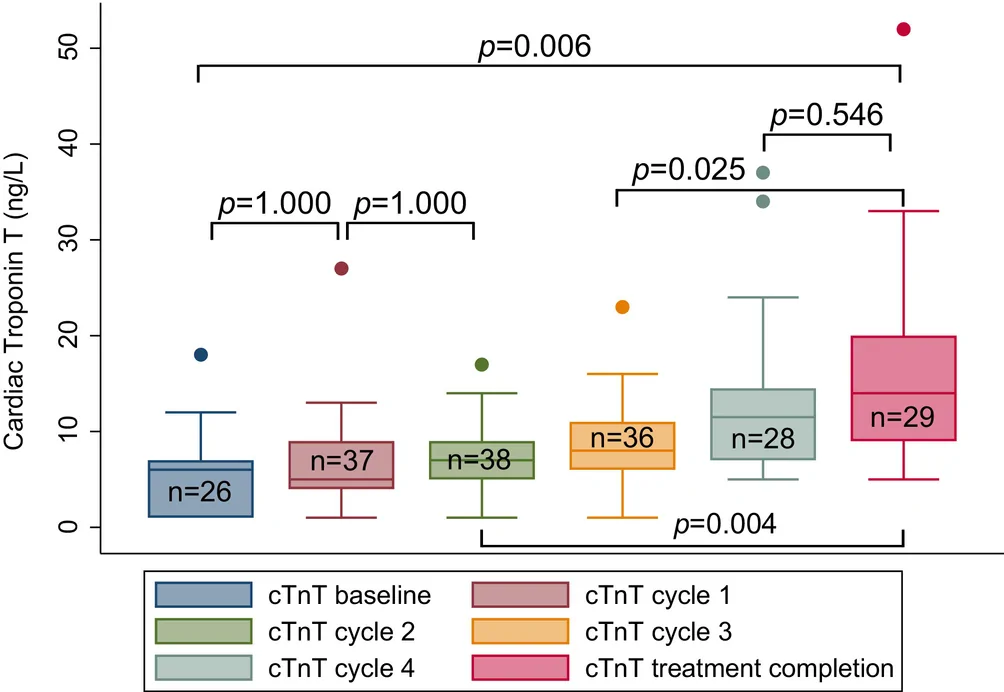
Increase in cardiac Troponin T between all timepoints. This figure shows the differences in cTnT between the timepoints. cTnT increases significantly between baseline and completion of therapy (*p* = 0.004). Significant differences were also observed for Cycle 2 vs. Cycle 5 (*p* = 0.004) and Cycle 3 vs. Cycle 5 (*p* = 0.025).

**FIGURE 5:**
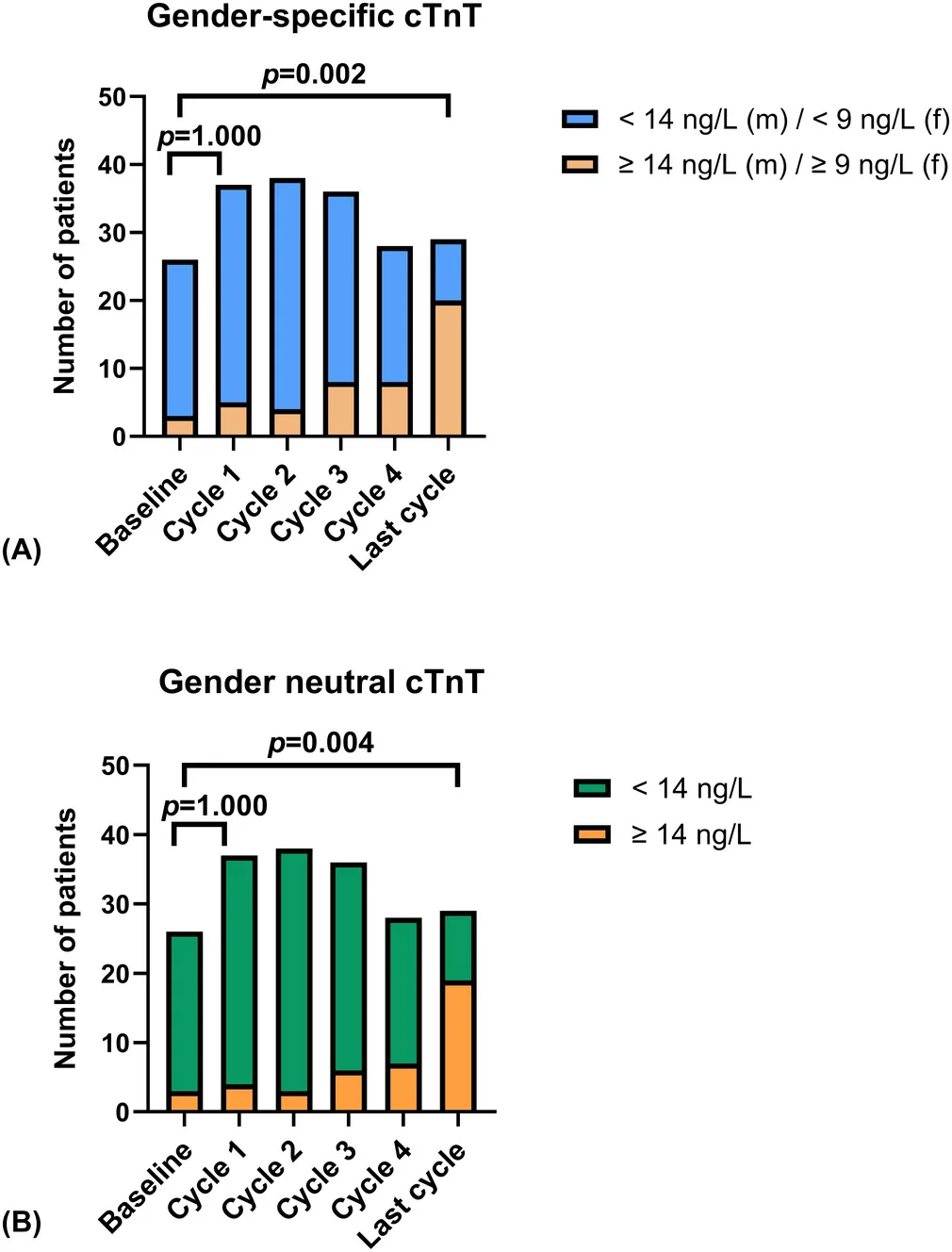
Elevated cardiac Troponin T. (A) Presents the number of patients with elevated sex specific cTnT (≥ 14 ng/L (m) and ≥ 9 ng/L (f)) at all timepoints. The number of patients with elevated cTnT increases significantly after completion of therapy compared to baseline. (B) Shows the same findings for a gender neutral cut‐off value of cTnT (≥ 14 ng/L). Last cycle refers to treatment completion.

### 3.4 Sensitivity Analysis

To assess the robustness of the primary findings, sensitivity analyses were performed using a complete‐case approach. For each pairwise comparison, only patients with data available at both relevant timepoints were included. The results from these complete‐case analyses were largely consistent with the primary analyses using mixed‐effects models. However, in the complete‐case analyses comparisons for GLS > −16% between baseline and short‐term as well as long‐term follow‐up and both RV strain between baseline and short‐term follow‐up reached statistical significance, whereas these comparisons were not significant in the primary mixed‐effects model.

### 3.5 Cardiotoxicity

The median follow‐up time from diagnosis of sarcoma was 2.4 [1.5–2.9] years. The primary study outcome of any grade of asymptomatic cardiotoxicity was observed in 30 patients (68.2%), of whom 2 (6.7%) were severe, 3 (9.7%) were moderate, and 25 (80.7%) were mild. Four patients with asymptomatic cardiotoxicity showed an LVEF of < 50% at short‐term follow‐up. In two of these patients, an ACE‐inhibitor was initiated for prevention of HF, after which the LVEF increased to a value within the normal range at long‐term follow‐up. In the two other patients with an LVEF < 50% at short‐term follow‐up, HF medication was not initiated due to palliative treatment of metastatic disease. All patients who developed any grade of asymptomatic cardiotoxicity showed a rise in NT‐proBNP at any timepoint, 21 patients (70%) had a rise in gender neutral cTnT, and 23 patients (76.7%) had a rise in gender specific cTnT. Patients who developed cardiotoxicity were treated with a significantly higher dose of anthracyclines (375.0 [375.0–450.0] mg/m^2^ vs. 337 [150.0–375.0] mg/m^2^, *p* = 0.017). Imaging‐defined CTRCD was observed in 5 patients (11.4%).

During follow‐up, 7 patients (15.9%) died, all due to tumor progression. Seven patients (15.9%) developed distant metastases, and in four patients (9.1%) relapse of the tumor occurred. Two patients (4.6%) were diagnosed with clinical heart failure (HF). One of these patients was admitted for HF, and consequently HF medication was initiated. In four other patients (9.1%) HF medication was also prescribed, in one patient as a result of clinical symptoms of HF.

### 3.6 Association Between Cardiac Biomarkers, Echocardiography and the Endpoints

Cox proportional hazards regression analysis was performed for the primary outcome of asymptomatic cardiotoxicity. In the univariable model, anthracycline dose per 75 mg increase (HR: 1.47 [1.03–2.10], *p* = 0.033) and increased gender neutral cTnT (≥ 14 ng/L) after Cycle 4 (HR: 2.86 [95% CI: 1.08–7.58], *p* = 0.034) were associated with development of cardiotoxicity. In the multivariable model, corrected for age, gender and anthracycline dose, increased gender neutral cTnT (≥ 14 ng/L) after Cycle 4 was still associated with development of cardiotoxicity (HR: 3.75 [1.28–11.0], *p* = 0.016). cTnT after completion of therapy was significantly associated with imaging‐defined cardiotoxicity (HR: 1.01 [95% CI: 1.0003–1.03], *p* = 0.044). Cox regression analysis was also performed for the secondary study endpoint of all‐cause mortality. In the univariable model, there was a significant association for baseline NT‐proBNP per 100 units increase (HR: 3.15 [95% CI: 1.11–8.93], *p* = 0.031), NT‐proBNP after Cycle 1 per 100 units increase (HR: 1.33 [95% CI: 1.01–1.74], *p* = 0.040) and baseline NT‐proBNP ≥ 125 ng/L (HR: 22.36 [1.39–359.03], *p* = 0.028). In the multivariable model corrected for age, NT‐proBNP after Cycle 1 per 100 units increase (HR: 1.33 [1.01–1.75], *p* = 0.046) showed a significant increased risk for death.

## 4 Discussion

We observed that systolic LV function as measured by LVEF and LV GLS significantly decreased after anthracycline chemotherapy in sarcoma patients. Both remained declined during short‐term follow up (6 months–1 year). The drop in LVEF and GLS occurred mainly between baseline and the end of treatment rather than after treatment termination. Our cohort showed a significant rise in cardiac biomarkers (NT‐proBNP and cTnT) between baseline and all cycles of chemotherapy. Two‐thirds of the patients in our cohort (68.2%) developed any grade of asymptomatic cardiotoxicity. Elevated gender neutral cTnT levels after Cycle 4 were associated with development of asymptomatic cardiotoxicity. We also observed an association between NT‐proBNP levels after Cycle 1 and increased risk of mortality. Our findings on cardiac biomarkers raise important questions that future large‐scale studies should address including the possible value of these markers to improve risk stratification.

### 4.1 Asymptomatic Cardiotoxicity

The number of sarcoma patients that developed mild, moderate, and severe asymptomatic cardiotoxicity in our cohort is consistent with prior literature [25]. Literature describes a dose‐dependent relationship between doxorubicin and cardiac dysfunction. This arises due to the generation of ROS, leading to cell apoptosis. In line with this, we found an association between the development of cardiotoxicity and treatment with higher doses of anthracyclines. Anthracyclines can cause a chronic reduction in LV function as assessed by GLS and LVEF. Early changes in GLS can be an initial sign of cardiotoxicity [26, 27]. Early detection of cardiac damage and early initiation of HF medication may prevent remodeling of the left ventricle and progression to (symptomatic) HF [28].

### 4.2 Echocardiography and Biomarker Surveillance Before, During and After Anthracycline Treatment

#### 4.2.1 Echocardiography

The ESC guidelines [29] recommend cardiac assessment with biomarkers and echocardiography in all patients planned for treatment with anthracyclines. Echocardiographic surveillance is advised after Cycle 4 (Class IIb) and 12 months after treatment (Class I). Measurement of cardiac biomarkers should be performed every two cycles and 3 months after treatment in low and moderate risk patients, who represented the majority of our cohort. Significant decrease of systolic and diastolic parameters was already observed at termination of treatment and did not significantly decrease thereafter. Therefore, our results support the guidelines to perform echocardiography shortly after anthracycline chemotherapy for early detection of subclinical (asymptomatic) cardiac dysfunction and timely initiation of HF medication to mitigate the development of clinical HF. LVEF is the most routinely used parameter to assess systolic function. However, a prior study in childhood cancer survivors has shown the presence of abnormal GLS in the absence of abnormal LVEF after anthracyclines. They also observed a relationship between high‐dose anthracyclines (≥ 300 mg/m^2^) and abnormal GLS. Therefore, GLS may have more clinical relevance in the prediction of cardiac dysfunction, especially in asymptomatic patients, compared to LVEF [30]. Measurement of GLS after initiation anthracyclines may, therefore, be of good prognostic value for future cardiotoxicity [9, 13].

#### 4.2.2 Cardiac Biomarkers

Cardiac biomarkers including NT‐proBNP and cTnT can assess the integrity of myocardial function and structure, respectively. Therefore, they could play an important role in diagnosing (subclinical) cardiotoxicity [31]. Prior studies observed abnormal biomarker values at any time during cancer treatment in almost 80% of the patients [25]. Research showed that NT‐proBNP increased in all patients after anthracycline therapy and that NT‐proBNP remained significantly increased until 1 year after chemotherapy [15]. In line with this, we observed elevated NT‐proBNP levels after at least one cycle of treatment compared to baseline, but there was no significant association between NT‐proBNP and LV dysfunction [14, 32].

The gold standard biomarker for the detection of myocardial damage is cTn as cTn is released after myocardial cell injury. Biomarker studies have observed an association between elevation of cTn and a greater reduction in LVEF and subsequent development of cardiotoxicity [28, 33]. In our study, cTnT increased significantly after baseline. Also, a significant increase in the number of patients with cTnT above predefined cut‐off values after completion of anthracycline therapy was observed. This pattern is consequent with prior literature reporting a significant increase of cTnI after anthracycline treatment [32, 34]. This increase in cTnT suggests that treatment with anthracyclines induces damage to the myocardial cells, which could lead to impairment of cardiac function. Moreover, cTnT levels above the predefined cut‐off value for gender neutral cTnT after anthracycline Cycle 4 and completion of therapy were associated with development of cardiotoxicity and imaging‐related cardiotoxicity, respectively. Cardiac biomarkers may be helpful in determining patients at risk for mortality and cardiotoxicity. We acknowledge that the gender distribution of our population is skewed and therefore we have analyzed gender specific as well as gender neutral cTnT cut‐off values.

### 4.3 Clinical Implications

A rise in NT‐proBNP can be caused by other factors, including cancer‐associated inflammation, renal function, and metastatic disease. Therefore, the utility of increased NT‐proBNP as a distinct biomarker to predict future cardiotoxicity can be questioned, which has also been described in the consensus statement by Plana et al. [28, 33]. Although NT‐proBNP was not associated with cardiotoxicity, NT‐proBNP at baseline and after anthracycline Cycle 1 was associated with all‐cause mortality. This association was also described by De Lullis et al. [15]. However, as the number of deaths in our population is low (*n* = 7), the effect size should be interpreted cautiously. We observed a significant increase in NT‐proBNP between baseline and Cycle 1, but levels did not significantly increase further in the subsequent cycles. As another study [15] also reported stabilization of NT‐proBNP, it could be useful to measure NT‐proBNP at baseline, after Cycle 1, and at termination of treatment. Following prior literature and our results, measuring cTnT at baseline and every two cycles thereafter may be helpful in assessing the risk of future cardiotoxicity. We would suggest using a gender‐neutral cut‐off value for cTnT in assessing the risk of cardiotoxicity.

In our cohort the majority of the patients had asymptomatic cardiotoxicity, but only two patients developed symptomatic HF. Although this number of patients (*n* = 2) is low, the percentage (4.5%) is in consistency with prior literature [35]. However, the prognostic value of asymptomatic cardiotoxicity on developing clinical HF should further be explored, given the uncertain prognostic trajectory of these subclinical abnormalities. Cardiac function in our patient population did not decrease any further between 6 months to 1 year after treatment, and our findings suggest that the incremental yield of performing an echocardiogram after 12 months may be limited if prior echocardiography did not show any clinically relevant abnormalities. However, given the significant decrease in LV systolic function before the end of treatment, performing an additional echocardiogram after two or three cycles of anthracyclines should be considered in high‐risk patients classified according to the guidelines as only performing echocardiography at the termination of treatment could be a missed opportunity for prompt intervention.

### 4.4 Future Perspectives

Currently, gender specific cut‐off values for NT‐proBNP are not reported in the cardio‐oncology guidelines. However, if these biomarkers would be more extensively used for screening purposes in the future, age and gender should be incorporated in defining risks. Our results suggest that cTnT is more accurate in predicting cardiotoxicity, whereas NT‐proBNP is a more general marker, which may be associated with mortality. However, the unique predictive value of these cardiac biomarkers needs further investigation and validation in larger cohorts to determine whether echocardiography should be routinely performed in all patients or if biomarkers can be used to identify high‐risk patients who should be evaluated with additional echocardiograms. Future studies on this topic should include prospective patient cohorts with longer follow‐up periods, more frequent echocardiographic assessments (including long‐term follow‐up), more consistently measured biomarkers and more extensive data on prophylactic treatments administered to patients with asymptomatic cardiotoxicity. This would allow for the assessment of long‐term cardiac remodeling, the persistence or resolution of functional changes. Consistent biomarker measurement enhances data comparability and strengthens the reliability of observed associations. All these improvements would strengthen the validity and interpretability of future findings.

### 4.5 Limitations

The retrospective nature of this single center study, relatively small sample size (*n* = 44), and the nonstandardized number of echocardiographic and biomarker measurements are main limitations of this study that may influence the generalizability of the findings. No a priori power calculation was performed, and given the small cohort size and number of parameters examined, this study is exploratory in nature and therefore the results should be interpreted as hypothesis‐generating. CTRCD defined according to guideline‐based criteria includes elevations in cardiac biomarkers as part of the outcome definition. As these biomarkers were also evaluated as predictors in the Cox regression analysis, incorporation bias cannot be excluded in these models, potentially leading to overestimation of the observed associations. However, analysis using imaging‐defined CTRCD was performed to mitigate this limitation. Further, the follow‐up period was relatively short, and due to the limited number of patients with clinical HF (*n* = 2), associations between echocardiography, cardiac biomarkers, and symptomatic CTRCD could not be established. Given the limited number of mortality, this analysis was restricted to an age‐adjusted model, which may result in residual confounding and limit the robustness and generalizability of the findings. Finally, as prophylactic treatment for HF was initiated in only a few patients, the sample size is too small to draw conclusions regarding the effect of this medication on echocardiographic parameters and cardiac biomarkers.

## 5 Conclusion

Sarcoma patients treated with high‐dose anthracyclines are at increased risk of developing LV dysfunction as assessed by LVEF and GLS in this population. Frequent biomarker and echocardiographic abnormalities were observed, and exploratory associations with mortality were noted. Both cTnT and NT‐proBNP levels increased significantly after one cycle of anthracyclines and remained elevated during treatment, underscoring the need for prompt monitoring and diagnosis for preemptive subclinical HF treatment. Interventional strategies or changes to guideline surveillance schedules cannot be defined from this study alone, and therefore future studies are needed to assess the unique predictive value of cardiac biomarkers in sarcoma patients in relation to echocardiographic screening to determine the need for echocardiography in all sarcoma patients undergoing anthracycline therapy.

## Author Contributions

**Elissa A. S. Polomski:** conceptualization, investigation, writing – original draft, methodology, visualization, writing – review and editing, software, formal analysis, project administration, data curation. **Frank M. Speetjens:** writing – review and editing, resources. **J. Wouter Jukema:** supervision, writing – review and editing, methodology. **Julius C. Heemelaar:** writing – review and editing. **Michiel A. J. van de Sande:** writing – review and editing, resources. **Hans Gelderblom:** writing – review and editing, resources. **M. Louisa Antoni:** conceptualization, methodology, validation, resources, project administration, supervision, writing – review and editing.

## Ethics Statement

The study was approved by the local institutional review board (protocol ID: 2023‐037) and complies with the Declaration of Helsinki.

## Consent

All patients provided written informed consent.

## Conflicts of Interest

The authors declare no conflicts of interest.

## Supporting information


**Table S1:** Definitions of asymptomatic CTRCD.
**Figure S1:** Diastolic dysfunction.

## Data Availability

The deidentified data from the Leiden University Medical Center that support the findings of this study and further information are available from the corresponding author upon reasonable request.
