# Supplementary material for: Asymptomatic Cardiotoxicity After High‐Dose Anthracycline Treatment in Sarcoma Patients Assessed by Biomarkers and Echocardiography
Source: Int J Cancer. 2026 Jun 30;159(11):2755–65. doi: 10.1002/ijc.70620 (PMC13637055; doi:10.1002/ijc.70620)
Supplement: Supplementary file 1 [file IJC-159-2755-s001.pdf]

Asymptomatic Cardiotoxicity After High-dose Anthracycline Treatment in Sarcoma Patients assessed by Biomarkers and Echocardiography

Elissa A. S. Polomski, Frank M. Speetjens, J. Wouter Jukema, Julius C. Heemelaar, Michiel A.J. van de Sande, Hans Gelderblom, M. Louisa Antoni

<u>Table of contents</u>

Supplementary table 1 - Definitions of asymptomatic CTRCD Page 2

Supplementary figure 1 – Diastolic Dysfunction Page 3

Supplementary Table 1. Definitions of asymptomatic CTRCD

**Severe asymptomatic CTRCD**	New LVEF reduction to < 40%[1]
**Moderate asymptomatic CTRCD**	New LVEF decline by ≥ 10% to an LVEF of 40-49%<br>**OR**<br>New LVEF decline by < 10% to an LVEF of 40-49% **AND EITHER**<br>1. New decline in GLS of > 15%<br>**OR**<br>2. New rise in cardiac biomarkers[2]
**Mild asymptomatic CTRCD**	LVEF ≥ 50%<br>**AND EITHER**<br>New decline in GLS of > 15%<br>**AND/OR**<br>New rise in cardiac biomarkers

1) A reduction from a normal LVEF (≥ 53%) to an LVEF < 40%. 2) A rise in biomarker levels between baseline and any timepoint to a value above the pre-defined cut-off value in the presence of a normal range at baseline or a rise beyond the biological and analytical variation of the assay used.

LVEF = left ventricular ejection fraction; GLS = global longitudinal strain.

Supplementary Figure 1. Diastolic dysfunction

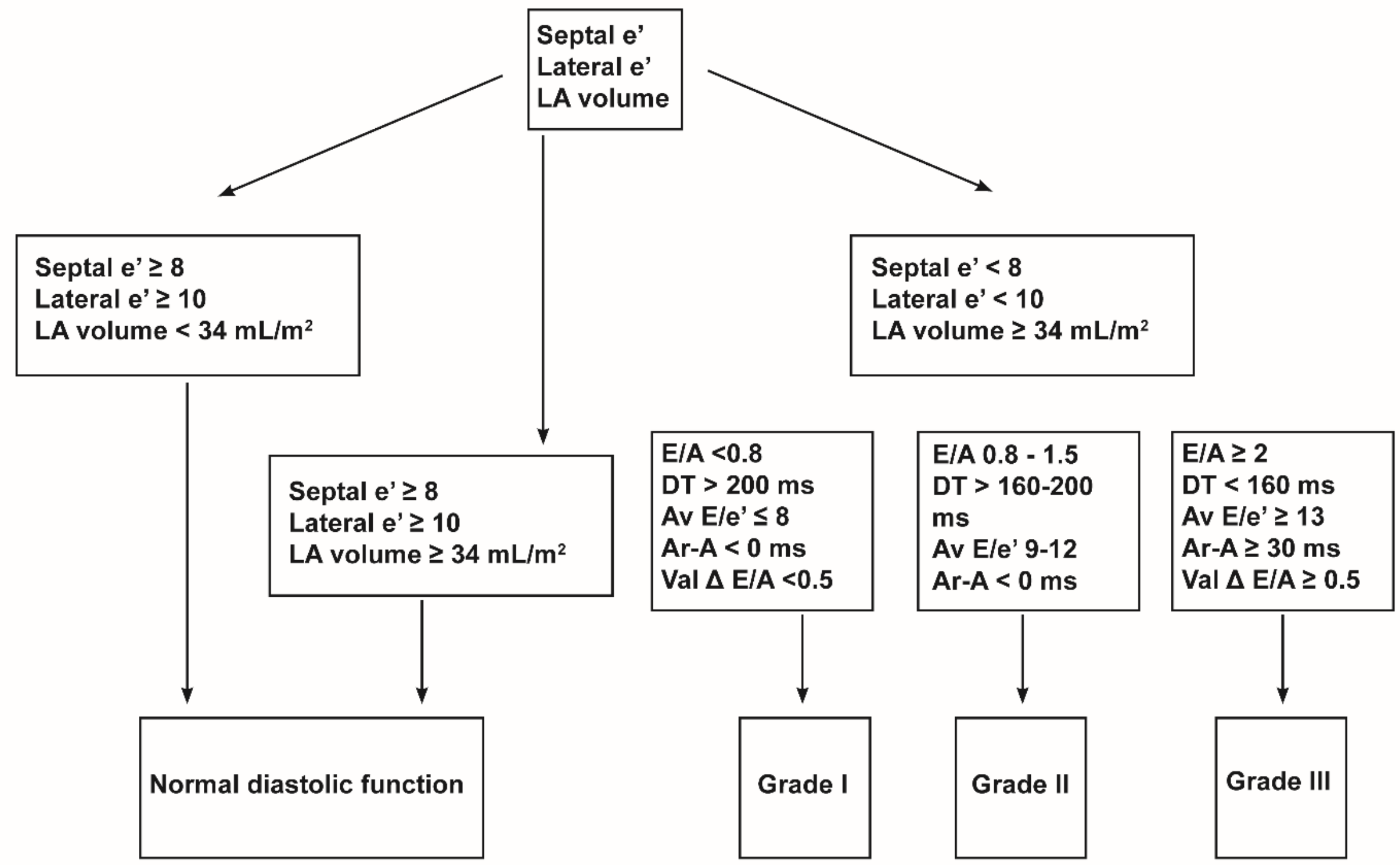

Septal e' = septal early diastolic mitral annular velocity; lateral e' = lateral early diastolic mitral annular velocity; LA = left atrial; E = early diastolic transmitral flow velocity; A = late diastolic transmitral flow velocity; DT = deceleration time; Av = average; Ar-A = Atrial reversal - mitral A wave interval; Val = change.

This figure was adapted from Nagueh et al., 2009.
